# Neurofeedback and meditation technology in outpatient offender treatment: a feasibility and usability pilot study

**DOI:** 10.3389/fpsyg.2024.1354997

**Published:** 2024-06-04

**Authors:** A. van der Schoot, J. Wilpert, J. E. van Horn

**Affiliations:** Research Departement, De Forensische Zorgspecialisten (DFZS), Utrecht, Netherlands

**Keywords:** feasibility, usability, neurofeedback, meditation, forensic outpatients, impulse control problems

## Abstract

**Introduction:**

Although Cognitive Behavioral Therapy (CBT) is the most often used intervention in forensic treatment, its effectivity is not consistently supported. Interventions incorporating knowledge from neuroscience could provide for more successful intervention methods.

**Methods:**

The current pilot study set out to assess the feasibility and usability of the study protocol of a 4-week neuromeditation training in adult forensic outpatients with impulse control problems. The neuromeditation training, which prompts awareness and control over brain states of restlessness with EEG neurofeedback, was offered in addition to treatment as usual (predominantly CBT).

**Results:**

Eight patients completed the neuromeditation training under guidance of their therapists. Despite some emerging obstacles, overall, the training was rated sufficiently usable and feasible by patients and their therapists.

**Discussion:**

The provided suggestions for improvement can be used to implement the intervention in treatment and set up future trials to study the effectiveness of neuromeditation in offender treatment.

## Introduction

Cognitive Behavioral Therapy (CBT) is viewed as one of the most effective psychological interventions to reduce re-offending (recidivism) and is widely implemented as evidence based forensic treatment in various forensic settings (Pearson et al., 2002; Landenberger and Lipsey, 2005; Wilson et al., 2005; Lipsey et al., 2007; Henwood et al., 2015). However, studies show that not all offenders benefit from CBT to the same extent (Babcock et al., 2004; Feder and Wilson, 2005; Eckhardt et al., 2013; Beaudry et al., 2021). It is known that certain offender characteristics, such as (comorbid) psychiatric disorders, can interfere with the success of treatment (Babcock et al., 2004; Eckhardt et al., 2013). Brazil et al. (2018) provide an explanation: effects of existing methods are mostly measured by self-reported behavioral outcomes such as aggression without an operationalization of the specific underlying constructs that contribute to offending behavior, which they consider detrimental for the effectiveness of offender treatment. They propose that biological (e.g., genetics, brain, and physiology) and cognitive functioning measures, and clinical observations would provide more insight into effectiveness of treatment programs in reducing recidivism rates. Also, incorporating biopsychosocial components should improve treatment effectivity by tailoring intervention techniques to specific perpetrator characteristics (Brazil et al., 2018; Beaudry et al., 2021).

CBT taps into cognitive and intellectual aspects and functioning, which are potentially less easily accessible to offenders with impulse control problems in particular. Impulse control problems impede the ability to foresee consequences, make achievable plans, choose from alternatives, control impulses, inhibit unwanted thoughts, and regulate social behavior (Heatherton and Wagner, 2011). Hence, impulse control problems are strongly related to the risk of general offending behavior (Moffitt et al., 2011; Loeber et al., 2012; Fergusson et al., 2013) and recidivism rates (Lloyd et al., 2014). As neurotechnology tunes into bodily mechanisms and experiential learning, as opposed to the cognitive methods which set out to finding explanations and alter thinking, it can be a valuable addition to offender treatment. Different neurotechnological methods have been developed to aid better self-regulation abilities, however, which of these methods are most suitable and effective to achieve effective treatment effects in offenders is yet unclear (Bijlsma et al., 2022).

As mentioned, in the search for treatment methods that are more effective, biopsychological factors should be taken into account (Brazil et al., 2018). Aggression can be the result of increased left frontal cortical activity (activity regarding approach) and decreased right frontal cortical activity (activity regarding inhibition) (Hortensius et al., 2012). It is therefore important to conduct research on interventions that work with left and right hemisphere asymmetry in aggression. Neurorehabilitation technology is an umbrella term for various technological applications and methods addressing specific brain functioning networks or pathways that are related to specific behaviors or symptoms. Some of these applications could yield promising prospects for offender treatment. For example, research suggests that both transcranial direct current stimulation (tDCS) and continuous theta burst stimulation (cTBS) are methods of neurorehabilitation that can play a role in the modulation of aggressive behavior by directly changing brain activity (Knehans et al., 2022). In a laboratory aggression task and questionnaire, Sergiou et al. (2022) demonstrated that HD-tDCS enhanced the frontal brain regions connectivity in a group of offenders, resulting in a decrease of aggressive responses. Subsequently, this could represent an innovative approach suitable for implementation in forensic outpatient treatment. However, HD-tDCS is a relatively expensive method which can only be administered by trained professionals. This renders it challenging for forensic outpatient clinics to offer this type of treatment. A cheaper and easier administrable method of neurorehabilitation, which has also been studied in offender populations, is EEG neurofeedback (Bijlsma et al., 2022).

Neurofeedback, also known as EEG biofeedback, is a technically supported form of real-time feedback of an individual’s brain activity through a brainwave monitoring device. In neurofeedback training, users learn to manipulate their neural activity based on direct feedback from the device. Since neurofeedback has been successfully used in treatment of impulse control problems in non-offenders (Sokhadze et al., 2008; van Doren et al., 2019; Hong and Park, 2022; Lima et al., 2022; Moreno-García et al., 2022) and is thought to target neural and cognitive processes that underlie offending behaviors (Bijlsma et al., 2022), it could also be a meaningful neurorehabilitation method in offender treatment (Van Outsem, 2011).

Although neurofeedback research in offender populations is scarce, initial results exhibit promise. Larson (2019) studied a small group of domestic violence perpetrators (*N* = 10) which received an intensive neurofeedback training. The treatment group (neurofeedback training) showed significantly lower Beta wave frequencies (e.g., active, alert, and focused mental states) than the control (no neurofeedback training) group. However, no significant differences were found between pre- and post-tests of participants’ self-reported feelings of anger, stress, and aggression. Furthermore, in a single case study on an adult with a history of sexual offending by Borghino et al. (2022), neurofeedback had a positive effect on control of sexual feelings, urges and behaviors. In yet another study on neurofeedback and recidivism, 20% of the treated incarcerated offenders (convicted of arson, sexual or violent offenses) had been rearrested, as opposed to 65% of the matched incarcerated offenders who did not receive neurofeedback (Von Hilsheimer and Quirk, 2006). More research is needed to fully understand how neurofeedback can contribute to offender treatment (Fielenbach et al., 2018).

Findings indicating a link between meditation practice and changes in brain regions and networks associated with impulsivity problems (Hölzel et al., 2011; Dambacher et al., 2015; Chaibi et al., 2023), suggest that neurofeedback combined with meditation, neuromeditation, could demonstrate an even larger positive effect on self-regulative behaviors, such as: attention regulation, body awareness, emotion regulation and change in perspective on the self (Hölzel et al., 2011; Sedlmeier et al., 2018). Since learning to enter calm states can be very challenging without feedback, insights into brain activity can facilitate to “get it right” in a more targeted manner. Through integration of real-time monitoring of brain waves and meditation practices, individuals can acquire the ability to swiftly enter a targeted state of brain relaxation and sustain this state over prolonged time (Tarrant, 2020). A benefit of neuromeditation in contrast to CBT, is that a patient can learn to (re)gain bodily and mind control in a top-down (internal self-direction) manner instead of bottom-up (self-direction through externally offered strategies).

An example of a neuromediation appliance is the Muse™ brain sensing wearable device. Via Bluetooth, the Muse EEG headband is connected to the Muse meditation app. It registers and recognizes Beta Waves (active, alert, and focused mental states) and Alpha Waves (relaxed and calm mental states) of the wearer and promotes Alpha states by providing auditory feedback (Muse, 2023).^1^ The presence of high Beta wave brain frequencies could hinder the ability to self-regulate emotions, as asynchronicity in frontal frequencies is related to aggression in offenders (Hortensius et al., 2012; Sergiou et al., 2022). Therefore, promoting Alpha states through neuromeditation with Muse could be a promising neurorehabilitation method supporting self-regulation in offenders.

Research showed that neuromeditation with Muse increased a state of mindfulness (ability to focus attention on the here and now, to feel less stress/tension) in adult participants in a non-clinical setting, represented by less mind restlessness and accurate attention to the breath. Participants reported the neuromeditation method to be an effective addition to their meditation practice (Hunkin et al., 2021a). Mindfulness has been shown to reduce impulse control problems (Gallo et al., 2021). Also, applied in general health care, Muse has demonstrated improvement of focused attention, reduction of physical symptoms, and supporting accelerated mindfulness learning. As a result, stress levels reduced and cognitive performance (such as faster reaction time and increased inhibition) improved (Bhayee et al., 2016; Taj-Eldin et al., 2018; Crivelli et al., 2019). Therefore, Muse EEG could be a beneficial technology in offender treatment. At the present, no documentation was found of prior studies involving the utilization of Muse neuromeditation technology within forensic settings.

### Central aim of the study

In preparation of a Randomized Control Trial (RCT) study, a pilot study was conducted to investigate the feasibility and usability of the Muse neuromeditation technology, in adult forensic outpatient treatment. It was expected that neuromeditation can be valued as a feasible and usable addition to treatment as usual.

## Method

The study was conducted in a Dutch forensic outpatient treatment facility between September 2022 and March 2023. An extensive test battery was applied using a multi-method design (self-report instruments, clinician-rated instruments, interrater-agreement, neuropsychological test, neuromeditation) at pre- and post-test (5 weeks after the pre-test) with weekly neuromeditation measurements. The research was approved by the Internal Review Board of the Van der Hoeven Clinic, indicating that it complies with the ethical guidelines of the institution and all laws and regulations in the Netherlands and Europe (2021-2-SC).

### Setting

Two locations of a Dutch forensic outpatient treatment facility were involved in the study. At these facilities, outpatients from the age of 12 receive treatment aimed at transgressive inclinations or behavior on a voluntary or mandatory base. Voluntary indicates that a patient enters treatment on their own initiative, on referral of a general practitioner or another mental health care professional. Mandatory treatment is imposed by a judge. Excluded for treatment are patients who are in acute psychiatric crisis, for example psychosis, severe addiction problems or suicidal tendencies. They are referred to the appropriate specialized mental health care.

Treatment for outpatients consists of a combination of various CBT elements, such as psychoeducation, self-monitoring, cognitive restructuring, improvement of coping skills or other evidence-based intervention techniques, such as Eye Movement Desensitization and Reprocessing (EMDR) and Acceptance and Commitment Therapy (ACT).

### Sample

Patients were eligible for participation if they were 18+ years of age and had at a score of 2 or higher on the dynamic risk factor “lack of impulse control” of the Forensic Outpatient Risk assessment and Evaluation (FORE V2; Van Horn et al., 2020; for more information, see Instruments section). A total of six female therapists, with a mean age of 28.83 years (*SD* = 3.8), registered to participate in the study and selected patients from their caseload who fitted the inclusion criteria.

Of the 11 patients enrolled in the Muse pilot study, eight completed the study: six males and two females with a mean age of 40.88 years (*SD* = 12.28, range 25–59 years). The three dropouts did not start with the neuromeditation sessions, because of other priorities such as treatment start-up, EMDR intervention and crises. The patients’ mean impulse control score on the FORE dynamic risk factor was 2.75 (*SD* = 0.71), ranging from 2 to 4. At the outset of their involvement in the study, participants had an average treatment duration of 8.38 months (*SD* = 5.98), with a range from 2 to 19 months. Table 1 presents several additional characteristics of the patients’ sample.

**Table 1:** Characteristics of the patients’ sample (*N* = 8).

	*n*(*%*)
Education	
Secondary vocational education	4(50)
Pre-secondary vocational education	2(25)
Primary education	2(25)
Primary diagnosis	
Disruptive, impulse-control and conduct disorders NOS	4(50)
Personality disorder	2(25)
Autism Spectrum Disorder	2(25)
Substance abuse	
Cannabis	3(38)
Cocaine/speed	2(25)
Binge drinking	1(13)
Reason to enter treatment	
Verbal and physical aggression	7(88)
Compulsive stealing	1(13)
Treatment context	
Voluntary	7(88)
Mandatory	1(13)

### 4-weeks neuromeditation training

At the start of each face-to-face session, patients received neuromeditation training using the Muse EEG headband (https://choosemuse.com; RRID:SCR_014418). In their study with Muse neuromeditation, Crivelli et al. (2019) concluded that a daily exercise of meditation (10–20 min) over the course of 4 weeks, resulted in positive effects. Following this, the study duration was set at 4 weeks. Therapists incorporated the neuromeditation training in the patient’s regular weekly treatment session, assisted by a protocol with a description of all the necessities and sequence of steps per session. Also, instruction manuals for both therapists and patients were provided with guidelines on how to use the Muse headband and mobile application (including the meditation exercises). More information on the Muse headband and mobile application is provided in the Instruments section.

Per session, 15 min were scheduled for the intervention, including pre- and post-inquiries and a brief evaluation. Each consecutive session, the headband wearing time was increased by 1 min, starting with 3 min in the first session. After the Muse intervention, the session was continued with treatment as usual. During the 4-weeks research period, patients were also asked to perform a daily mindfulness exercise at home.

### Instruments

The dynamic risk factor *D8 Impaired impulse control* of the risk assessment instrument *Forensic Outpatient Risk assessment and Evaluation* (FORE V2; Van Horn et al., 2020) was used as an inclusion criterion. This item measures out-of-control behavior or poor control over emotions, leading to disruptions in the past 6 months in daily life functioning at home, at work, during education, socially or financially. Patients were included if they scored 2 or higher (“some problems”) on a 5-point scale, with 0 “no impulse control problems” and 4 “severe impulse control problems in three or more life domains”. The risk assessment is conducted by therapists. From 2017, all forensic outpatient services are obligated to assess the recidivism risk of every person in mandatory forensic outpatient treatment. In 2019, the FORE was advised to use for treatment outcome monitoring and the prediction of risk in outpatient forensic care (Zicht op forensische zorg [View on forensic care], 2023). A study of the validity and reliability of the FORE V2 has yielded good results (Eisenberg et al., 2020).

#### Muse headband and mobile application

The Muse headband (S) and mobile application (Muse, 2023) were used for neuromediation (see text footnote 1). The headband measures patterns of frequencies in brain waves, which represent brain status (i.e., brain activity) using electroencephalography (EEG).

Muse translates these brain waves into real-time audio feedback on the Muse app. Feedback consists of three different weather sounds as a proxy of the user’s brain state. Users can choose between different soundscapes, such as rainforest (default) or beach. In the rainforest soundscape for example, sounds of storm and hard rain represent wandering thoughts, indicating that an individual is distracted, and attention is fluctuating (active mental state). Sounds of wind and soft rain, on the other hand, signal that the brain is in its natural state of rest. That is, the attention is not fluctuating, but there is no deep focusing either (neutral mental state). Lastly, dripping water and bird chirping convey a deep soothing focus (calm mental state). After a session, the Muse app displays the number of bird chirps “achieved” during the session and the percentage of calm mental state, as well as the number of “recoveries”, which mark the ability to reinstate from an active to a calmer mind. A study by Hunkin et al. (2021b) provided initial evidence for the internal consistency and validity of two Muse metrics (mean calm states and recoveries from active states) as indicators of state mindfulness.

Using the auditory feedback, the receiver learns what state the brain is in and how a calm brain state “sounds” and feels like. Mindfulness and meditation are used to control brain turmoil, for instance, by doing relaxation exercises or focusing attention (for example on breathing). The Muse app offers various guided and non-guided meditation exercises that can be used with or without the headband, such as a non-guided breathing exercise or guided relaxation exercises.

#### Feasibility and usability

Feasibility was defined as the degree to which the study protocol can be performed in a practical way in terms of study procedure and use of the Muse headband and app. Usability was defined as the degree to which a product is experienced as efficient and satisfactory by designated users to accomplish predetermined objectives within a defined usage context.

The evaluation forms (completed at post-test) to assess the feasibility consisted of several questions for therapists and patients, covering the feasibility topics: study protocol (e.g., comprehensible information, pre- and post-test experience), Muse headband (e.g., wearing comfort, easy to use), and Muse app (e.g., suitable exercises).

The usability was assessed using qualitative and quantitative information. Qualitative information consisted of feedback from therapists and patients on, for instance, perceived changes in relaxation skills and reduction of impulsivity. Some of the questions in the evaluation form were open-ended (e.g., about the pros and cons of the training), but most were dichotomous (yes/no) with a text field to optionally elaborate on the scoring. Furthermore, at the end of each neuromeditation session, patients were asked about their experiences with the Muse headband and the app.

Quantitative information was gathered per session and pre- and post-research period. The *Modified Overt Aggression Scale* (MOAS; Buitelaar et al., 2014) and a rating scale for bodily tension were administered in each neuromeditation session. The MOAS was used to assess patients’ level of occurred behavioral aggression in the past week. The MOAS is a check-off list to register incidents of aggression over the past week. The practitioner registers if any of the following aggression types occurred: verbal, physical, aggression against property and auto-aggression on a 5-point scale (0 “none” to 4 “frequent”). For example, for verbal aggression the scores represent 0 “no verbal aggression”, 1 “shouting angrily”, 2 “cursing viciously”, 3 “impulsively threatens violence toward others or self” or 4 “threatening violence toward others or self repeatedly or deliberately”. Subsequently, a higher score reflects a higher prevalence of aggression. The psychometric properties of the MOAS have been supported in earlier studies (Kay et al., 1988). Furthermore, therapists asked patients about the currently experienced *tension* in their body, mind, and breath directly before and after each headband usage, ranging from 1 “relaxed” to 10 “highly tense”.

Pre- and post-test changes in impulsivity, body tension, awareness, executive functioning, and aggression were measured with self-report instruments. Impulse control was measured with the *Barratt Impulsiveness Scale version 11* (BIS-11; Patton et al., 1995). The BIS-11 is a 30-item self-report questionnaire that measures impulsivity. Responses are given on a 4-point Likert scale (1 “rarely/never” to 4 “almost always”). High scores indicate a higher degree of impulsiveness. Since the original factor structure could not be confirmed in an adult forensic population (Ireland and Archer, 2008), we used the total BIS-11 score in this study. According to a review by Vasconcelos et al. (2012), the internal consistency (Cronbach’s α = 0.69 to 0.83) and test–retest reliability (correlation coefficient *r* = 0.66 to 0.83) of the BIS-11 were satisfactory in most studies.

Body awareness was measured with the *Anger Bodily Sensations Questionnaire* (ABSQ; Dutch: Zwets et al., 2014) and the *Dutch Scale of Body Connection* (SBC; Van der Maas, 2015). The ABSQ is a self-report questionnaire with 18 items about specific bodily sensations encountered when experiencing anger as a result of (perceived) provocation. Responses are given on a 5-point Likert scale (1 “not at all” to 5 “very much”). A higher score on the ABSQ indicates experiencing a higher amount of body signals when angry. In a study of Dutch offenders, the ABSQ showed good internal consistency (Cronbach’s *α* = 0.93) and test–retest reliability (*r* = 0.82; Zwets et al., 2014). The SBC is a 20-item self-report measure, designed to assess the subscales body awareness (12 items) and bodily dissociation (8 items). Items are based on common expressions of awareness of the body such as ‘I notice that my breath becomes superficial when I’m nervous’. Responses are given on a 5-point Likert scale (1 “not at all” to 5 “always”). Higher scores on the subscales represent more awareness/dissociation. The internal consistency of the subscales body awareness and bodily dissociation has been shown to be adequate (Cronbach’s *α* = 0.83 and 0.78 respectively; Price and Thompson, 2007).

To assess executive functioning (EF), the Dutch version of the *Parametric Go/No-Go Task* (PGNG; Langenecker et al., 2007; Dutch version: Van Horn et al., 2023) was used. The PGNG is a computerized task designed to measure cognitive flexibility (set shifting), response inhibition, and working memory. Validity studies demonstrate that the PGNG measures the core EFs in a psychometrically sound, brief, and ecologically valid way (Langenecker et al., 2007; Votruba and Langenecker, 2013). The task comprises three levels with increasing difficulty assessing attention, set shifting, and inhibitory control in levels 2 and 3. In all three levels, a series of letters is presented (x, y and z) at a fairly rapid rate. The aim is to follow certain rules as instructed, while responding to specific letters as quickly as possible by pressing the space bar. Outcome measures in the three levels are the percentage of correct target trials (PCTT, indicative of attention), and the percentage of correct inhibition trials (PCIT, indicative of inhibitory control, not assessed in level 1).

The short *Dutch version of the Aggression Questionnaire* (AVL-AV; Buss and Perry, 1992) was used to measure aggression. The 12-item AVL-AV is a self-report questionnaire and measures physical aggression, verbal aggression, anger, and hostility (with three items each). Items were scored on a 5-point Likert scale (1 “completely disagree” to 5 “completely agree”). Higher scores indicate more aggression, anger, and hostility. The AVL-AV shows good psychometric properties in aggressive offenders (Hornsveld et al., 2009).

The *System Usability Scale* (SUS; Brooke, 1996) was administered at post-test to quantify the overall usability of the Muse headband and app. The SUS consists of 10 items with scoring options on a 5-point Likert scale (1 “completely disagree” to 5 “completely agree”). The total score of the SUS can range from 0 to 100, with higher scores indicating better usability. Based on study mean quartiles, Bangor et al. (2008) consider usability scores from 52.01 as acceptable and from 72.75 as good. They found a good internal consistency of the SUS-items of Cronbach’s α = 0.85 and higher.

### Procedure

Therapists received documents from the research team containing information about the study content and procedures. The six participating therapists reached out to their patients (regardless of their treatment phase) when they met the inclusion criteria. Patients received an information leaflet and, after agreeing to participate, signed the informed consent form and filled out pre-test measures online, including several questions about their prior experience with relaxation exercises and motivation to participate in the study. This pre-test battery was accessed by an email link to Qualtrics (Version: March 2023),^2^ a secure online survey platform. The PGNG was administered under the guidance of the therapist at the outpatient facility. After the research period, post-tests were conducted with the same instruments following the same procedures. In addition, at post-test, both patients and therapists filled out an evaluation questionnaire via Qualtrics to assess the feasibility and usability of the study protocol and neuromeditation training.

### Strategy of analysis

Information from the pre- and post-tests (quantitative) and evaluation forms (partly qualitative) was analyzed using IBM SPSS version 27. Since this pilot study is not primarily aimed at quantifying effects, and the sample size is insufficient for generating statistically reliable insights into preliminary findings as well, any assertions or conclusions drawn from the data must be approached with caution. Instead, the presentation of frequencies and percentages, as well as averages and standard deviations, is undertaken solely for the purpose of providing a descriptive overview and to offer an insight into how these results contributed to the perception of the usability of the intervention. Regarding the qualitative data from the open-ended questions and possibility to elaborate on chosen answer categories, the following procedure was followed. Firstly, the first and second author independently rated the information in two main categories: ‘negative evaluation’ or ‘positive evaluation’. Statements from patients and therapists were categorized as negative if there were (to some degree) indications of problems and cons (e.g., too time consuming, unclear, difficult, without added value, etc.). Statements from patients and therapists were categorized as positive if there was (some degree of) evidence to the contrary, implicating no problems and pros. Secondly, Cohen’s kappa values were calculated for the feasibility (10 items) and usability (3 items) separately. Interpretation guidelines for Kappa values are as follows: *κ* < 0 = no agreement, 0.0–0.20 = slight agreement, 0.21–0.40 fair agreement, 0.41–0.60 = moderate agreement, 0.61–0.80 = substantial agreement and 0.81–1.0 = perfect agreement (Landis and Koch, 1977). Agreement on the feasibility items was moderate (*κ* = 0.55) and on the usability items fair (*κ* = 0.25). Following that, an agreement score was achieved per item, and these consensus scores are presented as the ultimate findings. Feasibility and usability were considered acceptable when at least 60 percent of the patients and therapists rated the items as positive, and good when 80 percent rated them as positive.

## Results

### Prior experience and expectations

Table 2 describes, among other things, the patients’ prior experience with relaxation exercise and their motivation and expectations entering the study. The two other motives to participate, as mentioned in Table 2, were to reduce stress levels and improve calmness. Patients expected their participation to result in increasing capability to relax and experience calmness, more bodily awareness and control and reduction of impulsive behavior.

**Table 2:** Pre-test survey questions patients (*N* = 8).

Question	*N*(%)
Prior experience with relaxation exercise	5(63)
Current practice of relaxation exercise	4(50)
Motivation participation study	
Learning relaxation exercises	3(38)
Improving capability to relax	7(88)
Body awareness could be an important addition to treatment	7(88)
Using the Muse headband	1(13)
Other	2(25)
Willing to perform daily relaxation exercise at home	8(100)
No difficulties with the use of English in Muse app	7(88)
Intention to plan an extra weekly session for Muse	5(63)

### Feasibility and usability as assessed by therapists

Information on the feasibility and usability of the study protocol and the neuromeditation as experienced by the therapists, is presented in Table 3.

**Table 3:** Feasibility and usability rating based on therapists’ experiences per patient (*N* = 8).

Topic	*n*(%)
**Feasibility**	
*Study protocol*	
Patients experience filling out questionnaires*	
No problems	5(63)
Some problems	3(38)
Patients experience performing PGNG*	
No problems	3(38)
Some problems	5(63)
Enough time (15 min) for Muse session	4(50)
Enough information in study protocol	7(88)
*Muse app and headband*	
Easy to use Muse app and headband	5(63)
**Usability**	
*Changes and added value of neuromeditation*	
Added value of Muse headband and app	7(88)
Observed changes in patient’s relaxation skills and/or impulsivity	7(88)
Inclined to use neuromeditation with other patients	8(100)

^*^Coded qualitative information by first and second author.

Table 3 shows that usability items were rated as good (>80%) by therapists and most of the feasibility items were acceptable (>60%), except for PGNG and session time which were rated below the acceptable threshold. The evaluation form responses provided by the therapists revealed several noteworthy findings. Among these, were the amount of questionnaires, and one patient’s challenges in interpreting certain items too literally. According to the therapists, patients experienced some frustration engaging in the PGNG task due to heightened difficulty levels. Furthermore, approximately half of the patients required more time (ranging from 20 to 25 min) during sessions to elaborate on their experiences with at-home meditation and the usage of the headband. Also, some patients experienced technical failure of the app, when pausing during an exercise.

Therapists were asked if patients indicated pros and cons of the Muse intervention. Therapists indicated that patients’ comments on feasibility were more often negative than positive (1 pro versus 10 cons). The drawbacks primarily centered around issues such as headband discomfort, lack of applicability of exercises, frustration due to not being able to change the weather, distraction by a foreign language (English to non-native speakers) during stress, much effort to perform exercises at home and uncertainty of the exercise’s impact as a result of the absence of feedback during home sessions. As for the usability, comments were more often positive than negative (11 pros versus 0 cons). The essence of the pros was that Muse provided patients with a straightforward method of acquiring relaxation skills and attuning to one’s bodily sensations.

Therapists reported 9 pros versus 10 cons regarding feasibility and 2 pros versus 0 cons concerning usability. As feasibility drawbacks, therapists mentioned several points of concern: equipment and procedure take time to get acquainted to, interference of the use of Muse when patients might prefer to discuss pressing matters first, complexity and inapplicability of specific Muse app exercises, additional time needed for session preparation (particularly during the pre-treatment phase which is frequently filled with diagnostic and working relationship-establishing activities).

In terms of feasibility advantages, therapists noted that Muse offers a structured approach with clear objectives, facilitates accessible feedback and learning, and appears to be more comprehensible for patients compared to merely receiving explanations. Specific relaxation moments within the treatment session also appeared to be beneficial.

As for usability, therapists observed that their patients exhibited increased calmness, reduced stress and impulsiveness, a shift towards “think first and act later,” and improved relaxation abilities. All therapists were inclined to use the Muse intervention again in the future with other patients, especially with problems such as stress, ADHD, trauma and sleeping difficulties.

### Feasibility and usability as assessed by patients

Table 4 summarizes the raters’ consensus of the evaluation form responses of the patients.

**Table 4:** Feasibility and usability rating based on patients’ experiences (*N* = 8).

Topic	*n*(%)
**Feasibility**	
*Study protocol*	
Comprehensible information	7(88)
Experience filling out questionnaires^*^	
No problems	6(75)
Some problems	2(25)
Experience performing PGNG^*^	
No problems	6(75)
Some problems	2(25)
Prior expectations met regarding participation in study	5(63)
*Muse app and headband*	
Clear explanation from therapists on how to handle Muse app	8(100)
At ease during headband session	6(75)
Headband comfortable	7(88)
Managed to do the daily exercise	2(25)
Suitable exercises in Muse app	7(88)
**Usability**	
*Changes and added value of neuromeditation with Muse*	
Muse contributed to earlier experience with mindfulness (*n* = 5)	3(60)
Muse contributed to changes in impulsivity	5(63)
Intention to use Muse/relaxation exercises in the future^*^	5(63)
	**M(SD)**
SUS score	70.94 (17.88)

^*^Coded qualitative information by first and second author.

As can be seen in Table 4, most feasibility topics were rated good (>80%). The only aspect patients considered unfeasible, was the daily meditation exercise at home. All usability items were rated acceptable (>60%).

Patients reported four pros and three cons regarding feasibility topics and one pro and zero cons on usability. Patients experiencing difficulties with the questionnaires, found them confronting and containing some hard questions. Also, two patients struggled with the PGNG. One of them retook the test (with better results) because of impeding fatigue during the first administration. The three patients indicating their expectations of participation were not met, emphasized that their experience exceeded their expectations in a positive way. Patients varied in their preferred soundscapes, some considered the rainforest sounds irritating and switched to another option. Performing a daily exercise at home proved to be a challenge for most patients because of a variety of reasons: difficulty finding a applicable exercise in the Muse app, uncertainty due to lacking Muse headband feedback, distraction from the environment and lack of daily structure to incorporate a regular time to exercise. In case patients found the Muse app exercises inapplicable, they resorted to YouTube for alternatives. Nevertheless, seven patients indicated that there were enough applicable exercises in the Muse app. The average rating (a report mark from 1 for very bad to 10 for very good) for the contribution of the home exercise in promoting relaxation was 4.25 (SD = 2.43, range 1–7). The report mark for the Muse headband session was 7 (SD = 1.69, range 5–10). Patients estimated that their optimal amount of time of headband usage would be 7 to 8 min. Three of five patients with prior experience with mindfulness, reported added value of Muse to this experience.

Regarding the usability, patients reported improved body awareness and control/ability to regulate peace of mind. Asked what they had learned from the Muse intervention, patients stated: discovering ways of breathing/thoughts to help calm down and relax, slow down thoughts, and build in moments of rest. Overall, patients’ feedback indicated they regarded the neuromeditation training feasible and usable, except for the perpetuation of the daily exercises at home. Also, the mean SUS-score of the sample indicated an acceptable, bordering good, usability of the Muse device and app.

### Usability: outcomes neuromeditation sessions

In Table 5, means and standard deviations of the within session measurements are presented. MOAS scores indicated no physical aggression towards others and some fluctuating auto-aggression, aggression towards object and verbal aggression towards others during the research period. All tension measures show a decrease after the headband usage. Participants encountered tension more frequently related to their bodies than their thoughts. The neuromeditation indicators, birds and recoveries, increased with every session, except for a drop in recoveries in the last session. Calm state remained similar during the first three sessions and increased slightly in the last.

**Table 5:** Within session assessment of prior aggression, current level of tension and neuromeditation results (*N* = 8).

	T1	T2	T3	T4
	M(SD)	M(SD)	M(SD)	M(SD)
**Aggression (MOAS)**				
Auto-aggression	0.25(0.46)	0.25(0.71)	0.88(1.25)	0.13(0.35)
Aggression objects	0	0.13(0.35)	0.50(0.76)	0.13(0.35)
Verbal others	0.88(0.84)	1(1.20)	0.75(0.89)	1.13(1.46)
Physical others	0	0	0	0
**Tension**				
General tension				
*Before*	4(1.51)	4(2)	5.25(2.38)	4.25(1.83)
*After*	3.13(1.13)	2.88(1.46)	3.88(2.30)	3.13(1.81)
Breath				
*Before*	2.38(1.30)	3.13(1.73)	3.88(2.59)	3.25(2.05)
*After*	2(0.93)	2.38(1.06)	2.75(1.98)	2.38(1.06)
Body				
*Before*	4.63(1.60)	4.88(1.89)	5.50(2.27)	4(1.93)
*After*	3(1.07)	3.50(1.31)	3.75(1.98)	2.75(0.89)
Thoughts				
*Before*	4.63(3.07)	4.13(2.23)	4.75(3.01)	4.63(1.69)
*After*	2.88(2.48)	3.38(2.67)	4.13(2.59)	3.25(1.98)
**Neuromeditation**				
Birds	5.50(4.75)	8.63(8.75)	14.38(15.34)	21.13(22.63)
Recoveries	2.38(2.45)	4.25(3.73)	7.13(8.90)	5(6.91)
Calm state	31.63(12.19)	30.75(19.86)	31.38(25.48)	35.25(28.29)

### Usability: outcomes pre- and post-test

Table 6 describes the means and standard deviations of the pre- and post-test outcome measures of the 4-weeks research period.

**Table 6:** Means and standard deviations of pre- and post-test outcome measures (*N* = 8).

	T1	T2
	M(SD)	M(SD)
*Impulsivity*		
BIS-11	72.38(7.29)	68.38(9.16)
*Body awareness*		
ABSQ	46.63(13.84)	45(11.07)
SBQ Body awareness	3.35(0.42)	3.33(0.47)
SBQ Dissociation	3.06(0.49)	2.81(0.48)
*Executive functioning*		
PGNG Task PCTT	77.49(17.50)	79.21(15.20)
PGNG Task PCIT	66.01(28.54)	68.55(29.86)
*Aggression*		
AVL-AV Physical	11(3.16)	9.38(3.74)
AVL-AV Verbal	8.75(1.28)	7.25(0.89)
AVL-AV Anger	9.63(2.20)	9.63(2.50)
AVL-AV Hostility	9.88(3.56)	9.38(3.20)

Due to the small sample size, pre- and post-test results can only be compared on face value, see Table 6. Impulsivity, body awareness and aggression decreased, except for the aggression subscale anger, which remained the same. Executive function increased with more correct and inhibition trials.

## Discussion

In this multi-method pilot study, the primary goal was to assess the feasibility and usability of the research protocol of a neuromeditation training offered to forensic outpatients. This neuromeditation training was introduced as an additional intervention to the usual treatment (primarily CBT), using the integrative neurofeedback and meditation technology of Muse. Six therapists (all females) and eight patients (6 males and 2 females) participated in the study.

The feasibility and usability of neuromeditation were evaluated through participants’ feedback, providing insights into its practicality and perceived benefit. Patients expressed motivation to engage in neuromeditation to enhance relaxation and bodily connectedness. Notably, patient expectations were either met or exceeded following the neuromeditation training, corresponding with findings from Hunkin et al. (2021a) regarding mindfulness experiences.

Both therapists and patients acknowledged the study protocol’s comprehensiveness. However, both also voiced a preference toward lengthier and more frequent neuromeditation sessions. Patients suggested an optimal session duration of 7–8 min, in agreement with the findings of Hunkin et al. (2021a) who reported favorable effects from 7-min neuromeditation sessions. Furthermore, Hunkin and colleagues underscored the potential benefits of introducing a training phase for app familiarity and auditory feedback to overcome performance hindrances caused by stress. The inclusion of a training phase holds particular promise, considering that participants in future studies might have less prior meditation experience than the current sample.

In terms of questionnaire relevance, it became apparent that not all measures were equally pertinent. The absence of improvements in the Modified Overt Aggression Scale (MOAS), might suggest that aggression is an unsuitable outcome measure of the neuromeditation intervention. This aligns with patients being in forensic treatment not only for aggression but also for other issues, such as compulsive stealing. The common denominator was impulse control problems. Brazil et al. (2018) also emphasize that in identifying appropriate treatment methods for offenders, the underlying biopsychosocial constructs are better indicators of treatment applicability than the operationalization of aggression through behavioral constructs. Hence, using the MOAS to operationalize aggression may also be inadequate for this purpose.

Furthermore, the lack of applicability of exercises in the Muse app could be addressed by consulting patients about exercise preferences and offering alternatives from established treatment protocols (e.g., Re-Art, Hoogsteder and Bogaerts, 2018) or external resources like YouTube. Additionally, collaborative planning for at-home practice, considering suitable settings and timing to minimize disruptions during exercises, emerged as an important strategy to address challenges. Selecting an appropriate phase for the introduction of the intervention is also vital; one therapist noted that the pre-treatment phase might not be the most suitable time due to other priorities. The dropout of three of the 11 enrolled patients further emphasizes the significance of appropriate timing to prevent disruption in treatment progress.

Therapists had the impression that five out of eight patients struggled with the cognitive executive function task, while only two patients self-reported difficulties. This discrepancy in perspectives might be attributed to therapists’ professional focus on patients’ behaviors observed in sessions. In contrast, patients likely evaluated based on a wider range of situations, including those not discussed or observed during the sessions. Both perspectives, one not necessarily more reliable than the other, contribute to a more complete picture. These divergences only underscore the necessity of acknowledging and discussing both therapist and patient perspectives in treatment. In this study, therapists’ and patients’ agreement in wishing to continue neuromeditation in regular treatment, reflects their shared acknowledgment of its supplementary value.

### Usability

At first glance, improvements were evident across all self-report measures, except the anger scale of the aggression questionnaire, and the executive functioning task. Although no statistical analyses could be performed due to small sample size, patients reported experienced improvements in overall impulsivity problems after neuromeditation. This substantiated in modest yet impactful enhancements such as improved performance in achieving “calm” states during neuromeditation training, reduced overall tension post-neuromeditation, lowered scores in impulsivity and aggression-related metrics (excluding anger), heightened body connectedness, and augmented inhibitory control performance in neuropsychological tasks. Therapists also corroborated these positive changes, observing heightened calmness, diminished stress, reduced impulsiveness, and increased relaxation capacity in patients. Notably, therapists expressed more optimism regarding changes than patients, indicating improvement in seven of eight patients, whereas five of eight patients acknowledged experiencing positive changes.

Based on the motivational direction model of frontal asymmetry in which relative left frontal cortical activity is associated with approach motivation (Harmon-Jones, 2003), there is a risk that neuromeditation could also suppress positive behaviors such as social behavior. Various studies show that meditation promotes social behaviors (Engert et al., 2023). There is however no research that takes into account the effects of neuromeditation on social behavior. Future studies should look at a range of emotional and social behaviors to better understand the full impact of the treatment.

Although this was primarily a feasibility and usability study, and we cannot conclusively determine its effectiveness, the positive results observed in some patients are noteworthy. These results across various measures indicate the potential benefits of combining meditation with neurofeedback.

### Strengths and limitations

One of the notable strengths of this pilot study lies in its pioneering approach, integrating neuromeditation technology into the treatment of forensic outpatients with impulse control problems. The study’s emphasis on user experience and feedback from both therapists and patients provides valuable insights into the feasibility and usability of this innovative intervention for offenders.

However, several limitations should be acknowledged. First and foremost, the small sample size restricts the generalizability of the findings. The participants were characterized by their familiarity with substance use (75%) and meditation (63%). Individuals with substance use problems have shown to exhibit higher impulsivity than non–substance-users (Moeller and Dougherty, 2002), which is a concern in forensic treatment since it poses a risk for violence in offenders (Pickard and Fazel, 2013). These factors might have resulted in overrepresentation of these characteristics in our study sample. The presence of previous meditation experience might have positively impacted motivation, potentially lowering the reluctance to participate. Conversely, individuals lacking familiarity with meditation might be less inclined to engage with novel meditation-based interventions. This selection bias limits the extent to which the results can be extended to a broader range of forensic patients. Additionally, the relatively short duration of the intervention (4 weeks) and relatively little headband usage may have hindered the emergence of more pronounced changes in measured outcomes. A longer intervention period might be necessary to observe substantial alterations in behaviors and self-regulation. Another aspect requiring attention pertains to the length and format of the pre- and post-intervention assessment battery. While the inclusion of such measures is crucial for evaluating the outcomes, the potential burden of lengthy questionnaires and evaluation forms should not be underestimated. To enhance participant engagement and minimize response fatigue, these assessment tools should remain concise, focused, and pertinent to the objectives at hand.

A final disadvantage, is that as non-developers we have little insight into Muse’s headband and app specifications and therefore lack certain information to get a better idea of its working mechanism processes. For the benefit of effect studies, this would need further investigation. To enhance the effectiveness of studies, further exploration is necessary. Future research should consider how technological factors, like the exact placement of EEG electrodes, relates to treatment effectiveness for impulse control problems in offenders.

### Clinical implications

The outcomes of this pilot study hold clinical implications for the treatment of forensic outpatients with impulse control problems. Neuromeditation, as facilitated by the Muse technology, offers a unique avenue for enhancing self-regulatory skills and reducing impulsivity. By focusing on real-time monitoring of brain activity and coupling it with meditation practices, individuals can gain the ability to access and maintain desired states of relaxation. The encouraging feedback from both therapists and patients, indicating positive changes in relaxation skills and reductions in impulsivity, highlights the potential of this approach. The study’s findings underscore the need for a nuanced understanding of intervention timing within the treatment process. The introduction of neuromeditation might be most effective when patients are not overwhelmed with other treatment priorities, allowing them to fully engage with the intervention. The varied experiences of the study’s participants point to the importance of tailoring neuromeditation exercises to suit individual preferences, and ensuring that patients can incorporate them into their daily routines.

## General conclusion

In conclusion, this pilot study ventures into the realm of neuromeditation as an innovative method for enhancing self-regulation and reducing impulsivity among forensic outpatients. The results suggest that neuromeditation, implemented through the Muse technology, holds promise in employing this intervention in this population. While the study’s small sample size and short intervention duration warrant caution in drawing conclusions, the findings provide valuable insights into the feasibility and usability of this approach. The positive feedback from both therapists and patients, coupled with the observed improvements in relaxation skills and reductions in impulsivity, point towards a potential value of neuromeditation as a supplementary treatment modality.

This study lays the foundation for future research endeavors in this domain, emphasizing the importance of larger-scale studies to evaluate the impact of neuromeditation on forensic patient outcomes. By addressing the methodological limitations and incorporating the suggestions for protocol improvement, further investigations can shed light on the true potential of neuromeditation in augmenting offender treatment. While the findings are preliminary, they signal a promising direction for advancing the field of forensic psychology through the integration of neuroscientific knowledge and innovative technologies.

## Data availability statement

The raw data supporting the conclusions of this article will be made available by the authors, without undue reservation.

## Ethics statement

The studies involving humans were approved by the Internal Review Board of the Van der Hoeven Clinic, indicating that it complies with the ethical guidelines of the institution and all laws and regulations in the Netherlands and Europe (2021-2-SC). The studies were conducted in accordance with the local legislation and institutional requirements. The participants provided their written informed consent to participate in this study. Written informed consent was obtained from the individual(s) for the publication of any potentially identifiable images or data included in this article.

## Author contributions

AS: Conceptualization, Data curation, Formal analysis, Investigation, Methodology, Project administration, Resources, Validation, Visualization, Writing – original draft, Writing – review & editing. JW: Data curation, Formal analysis, Methodology, Supervision, Writing – original draft, Writing – review & editing. JH: Methodology, Project administration, Resources, Supervision, Writing – review & editing, Conceptualization, Formal analysis, Validation.

## Footnotes

^1^https://choosemuse.com

^2^https://www.qualtrics.com
